# Supplementary material for: Prickle isoform participation in distinct polarization events in the Drosophila eye
Source: PLoS One. 2022 Feb 11;17(2):e0262328. doi: 10.1371/journal.pone.0262328 (PMC8836327; doi:10.1371/journal.pone.0262328)
Supplement: S1 Fig — (PDF) [file pone.0262328.s001.pdf]

original image for Fig 7A, anti-Pk[C]

1: w1118
2: pk^{30}
3: sple
4: pk-$sple^{13}$
5: Act5c > pk (UDRC)
6: Act5c > GFP-sple

eye discs

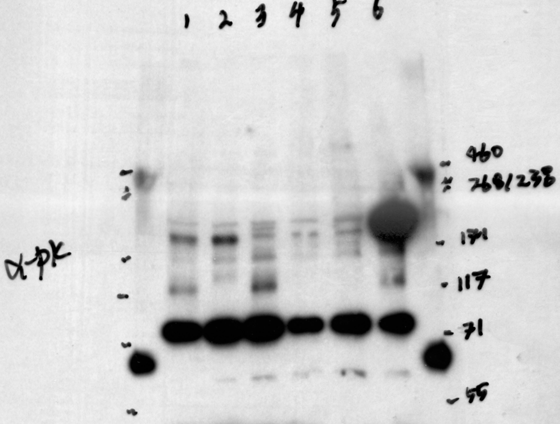

original image for Fig 7B, anti-Arm

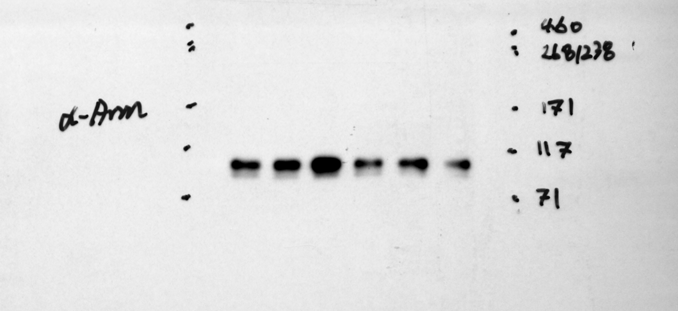

original image Fig 7B, anti-Pk[C]
36 and 40 hours

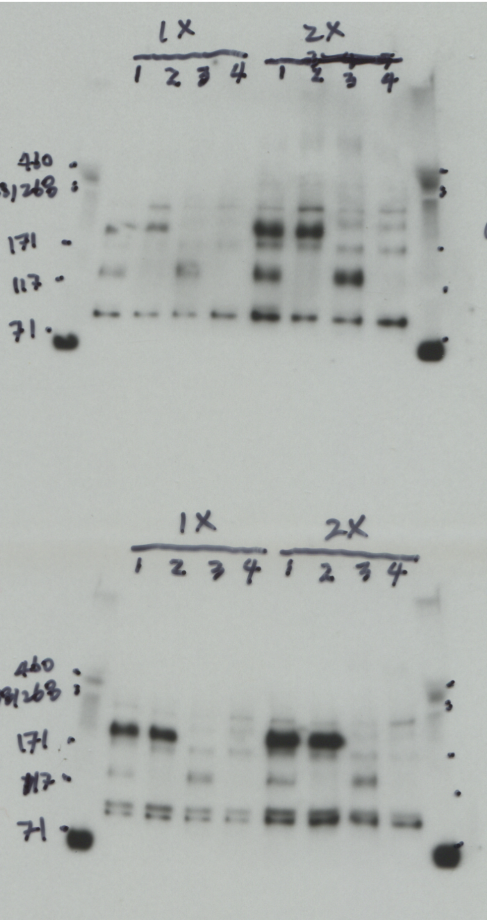

original images Fig 7B, lower portions of filters cut and probed with anti-tub.

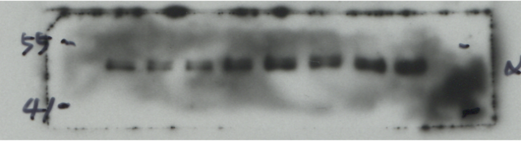

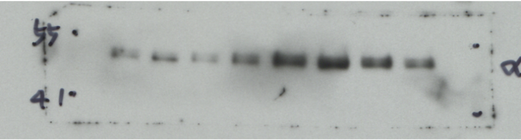

original image Fig 6-2B, cut and upper portion probed with anti-Pk[C], lower with anti-tub

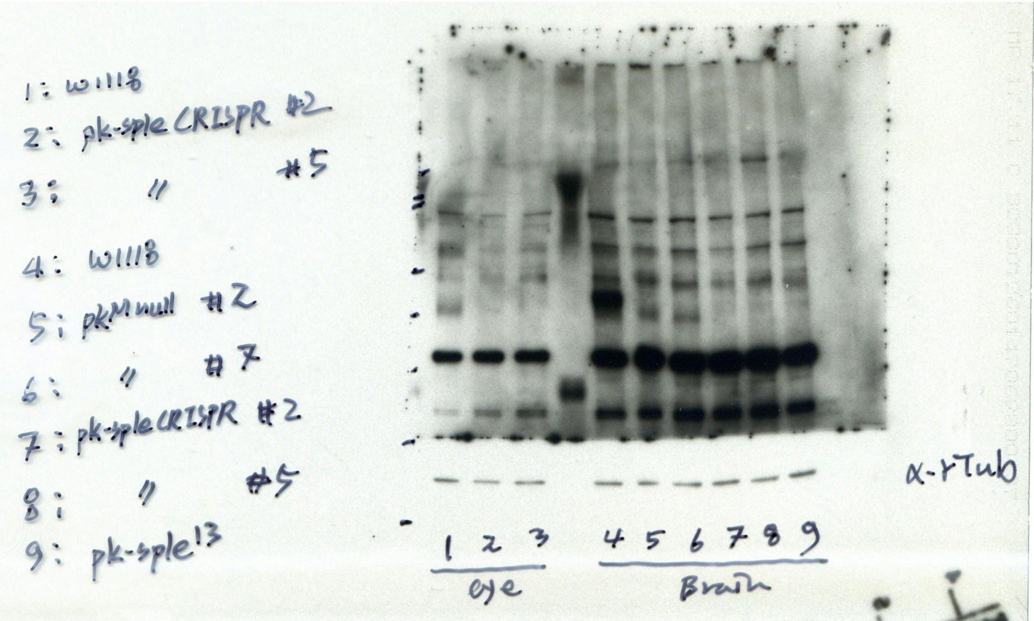

original image Fig 6-2C, probed with anti-HA. Lane marked with red X expressed a smaller HA-tagged protein as a control and was cropped out

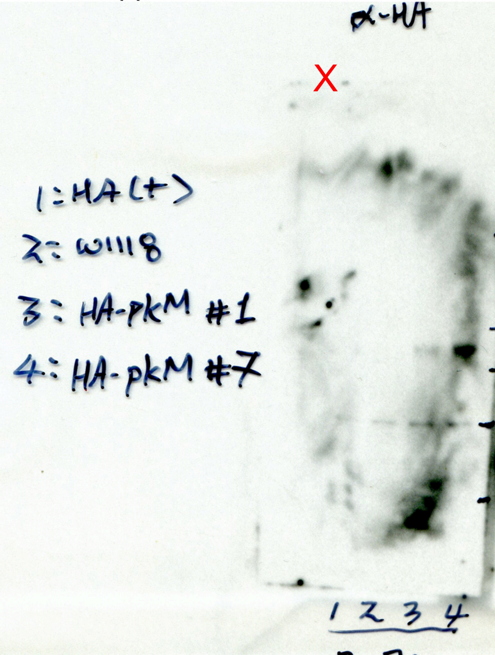

1: HA(+)
2: w1118
3: HA-pkM #1
4: HA-pkM #7

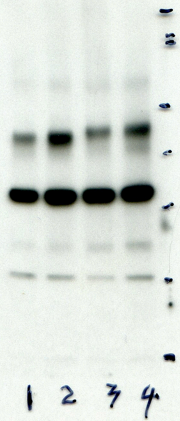

original image Fig 6-2C
probed with anti-Pk[C]
