# Supplementary figures and images for: Prickle isoform participation in distinct polarization events in the Drosophila eye
Source: PLoS One. 2022 Feb 11;17(2):e0262328. doi: 10.1371/journal.pone.0262328 (PMC8836327; doi:10.1371/journal.pone.0262328)

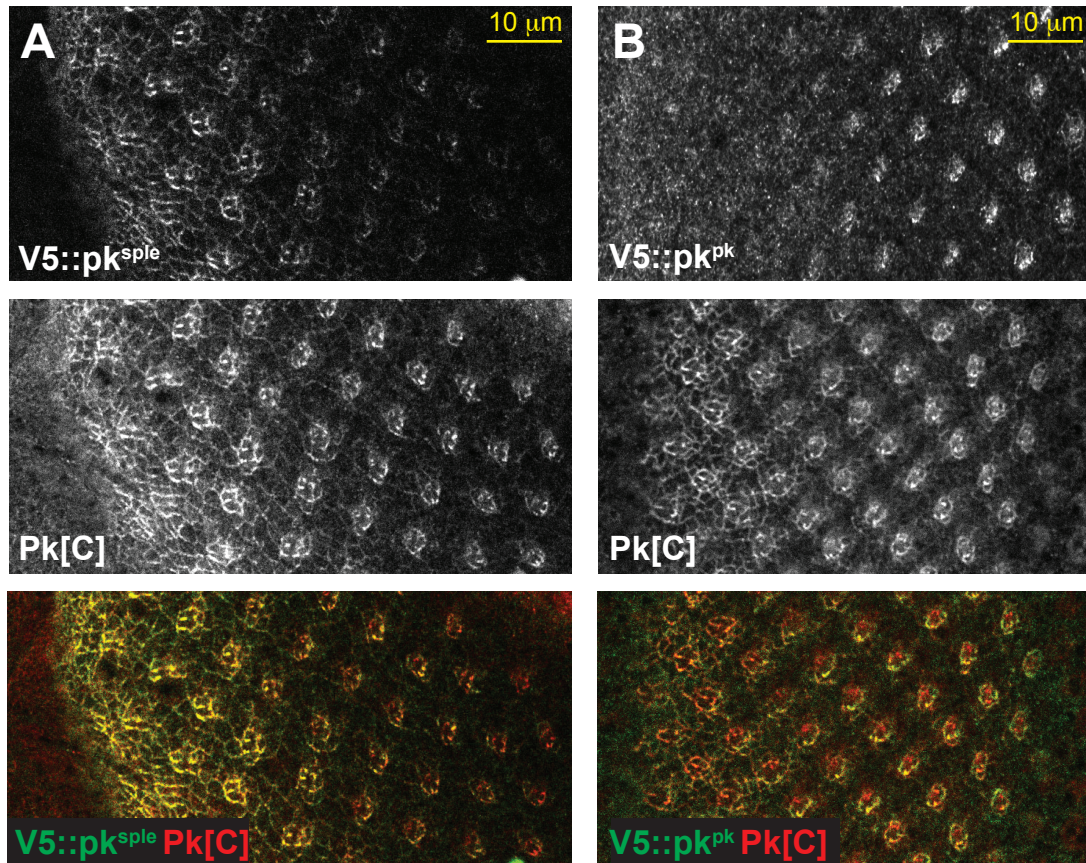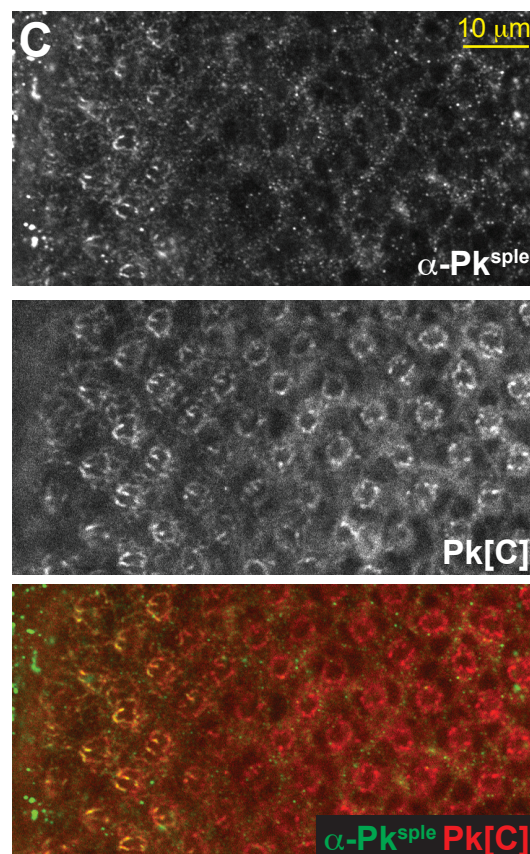

Supplement: S2 Fig — V5::Pksple (A) and V5::Pkpk (B) eyes co-stained with the common Pk antibody Pk[C]. The common antibody detects signal in apico-lateral junctions of all ommatidia, whereas V5::Pksple reports signal selectively in apico-lateral junctions of young ommatidia and V5::Pkpk reports signal selectively in apico-lateral junctions of older ommatidia. C. Control eye co-stained with anti-Pksple and Pk[C]. The anti-Pksple antibody detects early ommatidia, similar to V5::Pksple. (PDF) [file pone.0262328.s002.pdf]

Supplemental Figure 3-1

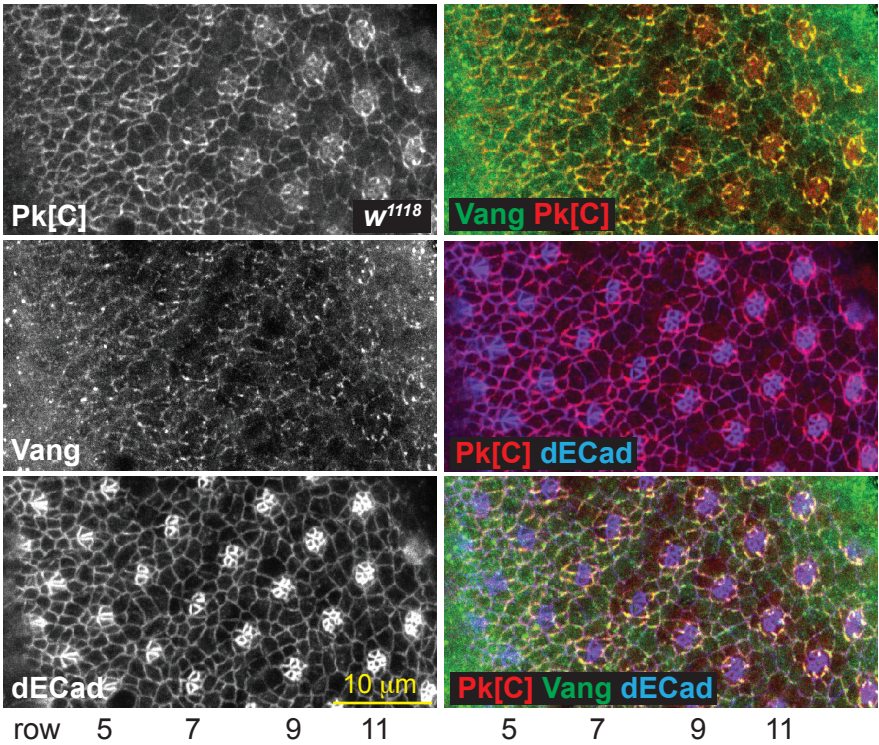

Supplement: S3 Fig — A control (w1118) eye stained with common Pk[C] (red in merge), anti-Vang (green in merge) and anti-dECad (blue in merge) antibodies. Vang co-localizes with all Pk isoforms in the apico-lateral junctions of ommatidia. (PDF) [file pone.0262328.s003.pdf]

Supplemental Figure 3-2

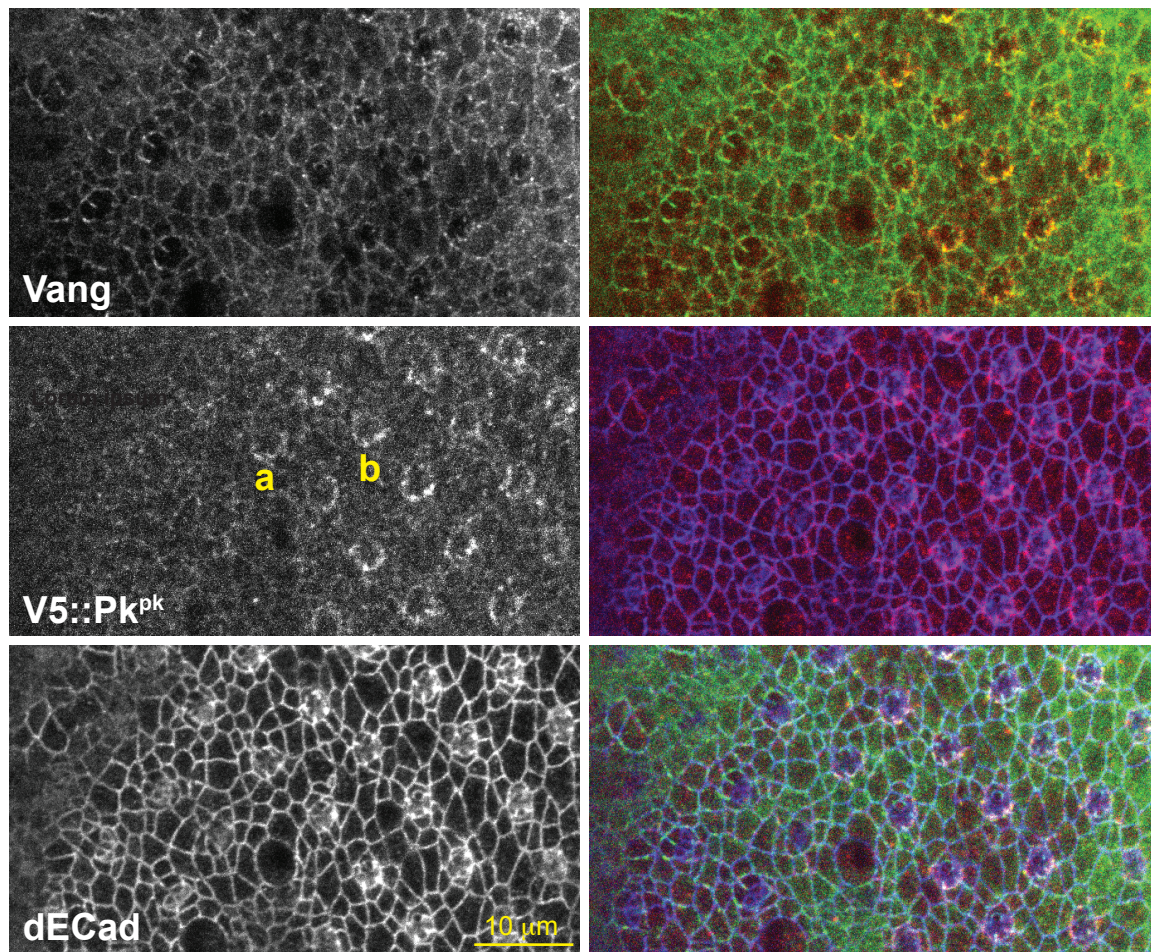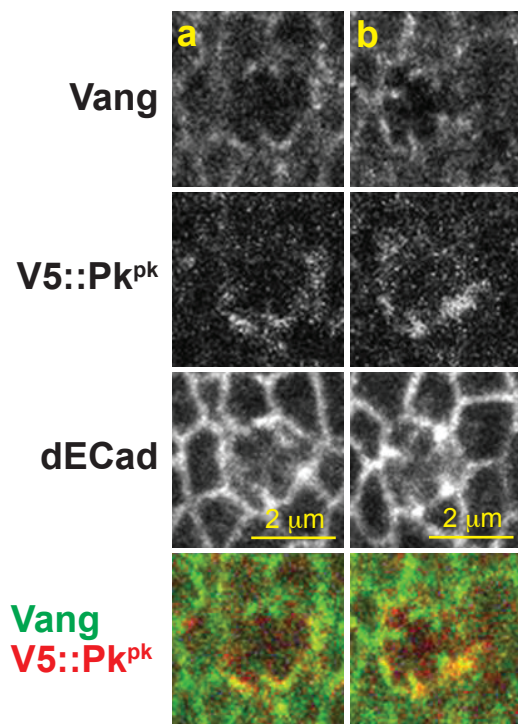

Supplement: S4 Fig — A V5::pkpk eye stained with anti-V5 (red in merge), Vang (green in merge) and dECad (blue in merge) antibodies. Vang co-localizes with Pkpk in the apico-lateral junctions of older ommatidia. (PDF) [file pone.0262328.s004.pdf]

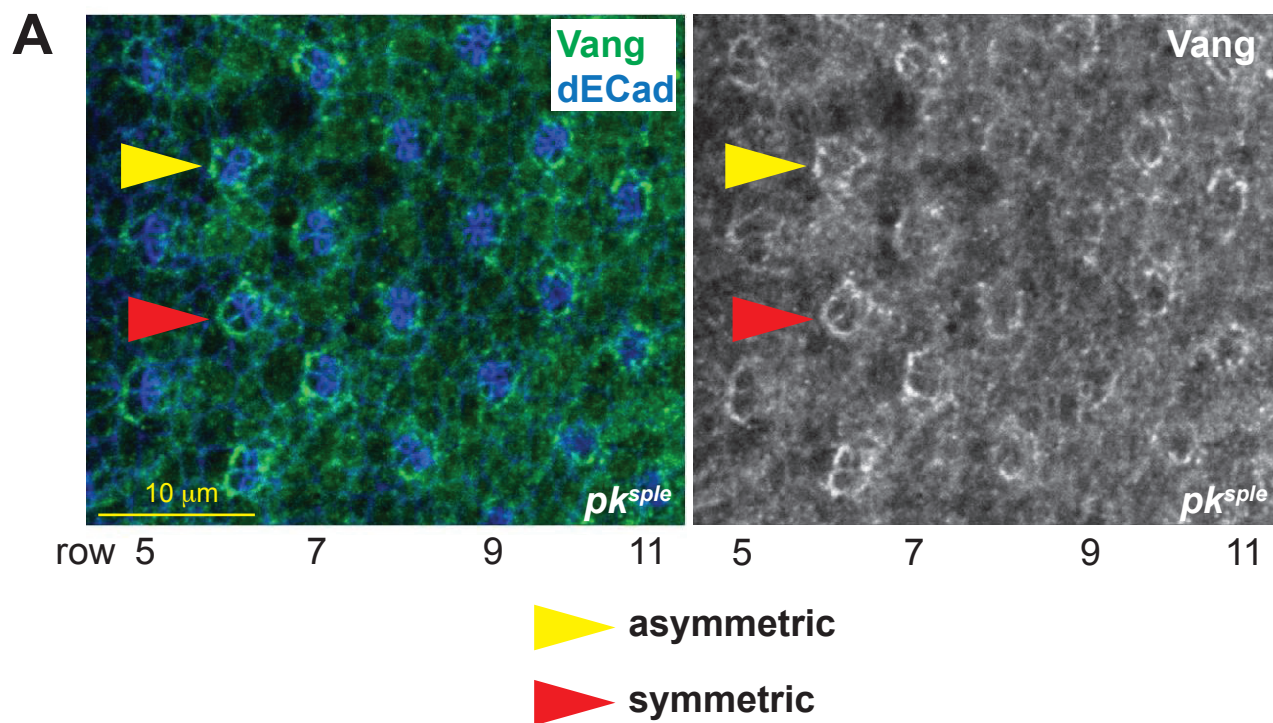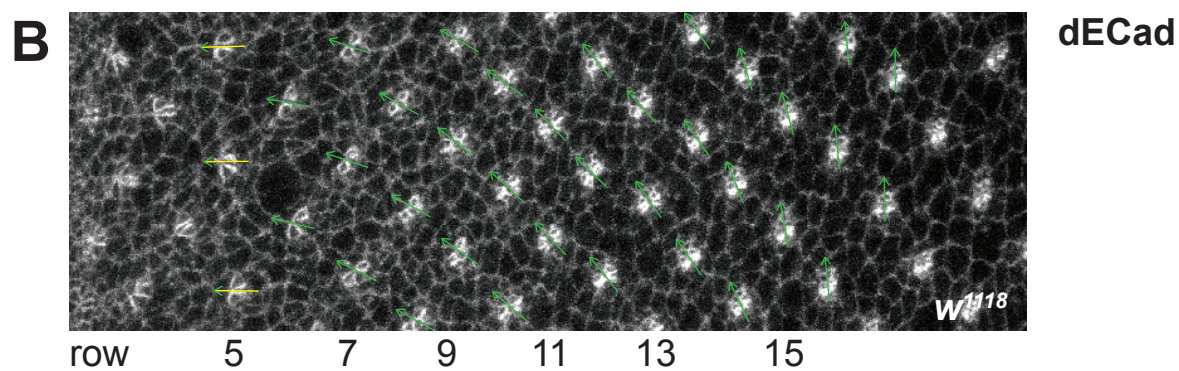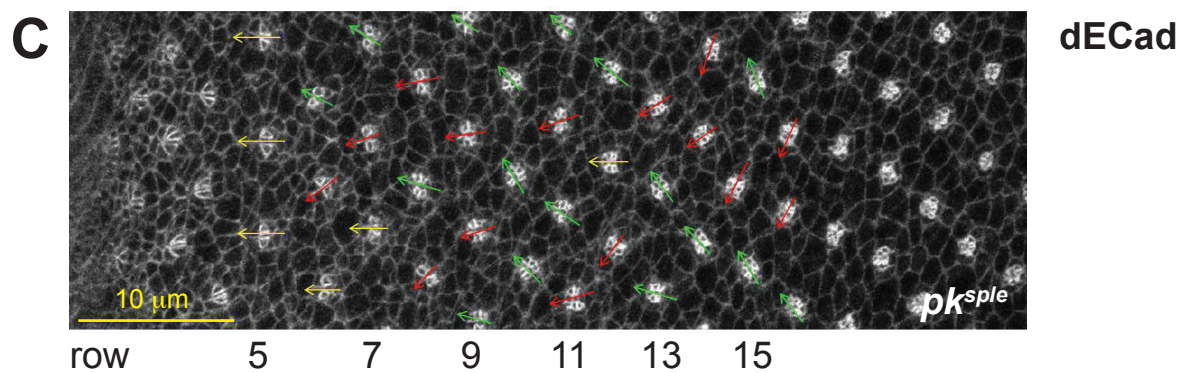

Supplement: S5 Fig — A. A pksple eye stained for Vang (green) and dECad (blue) showing asynchronous acquisition of molecular asymmetry. Two row 6 ommatidia are labeled, one showing Vang asymmetry (yellow) and the other showing symmetric distribution. B. A control (w1118; B), and C. a pksple mutant eye stained for dECad with approximate orientations of ommatidia shown with arrows. While rotation is approximately synchronous in the control eye (B), rotation is highly asynchronous in the pksple mutant eye (C). (PDF) [file pone.0262328.s005.pdf]

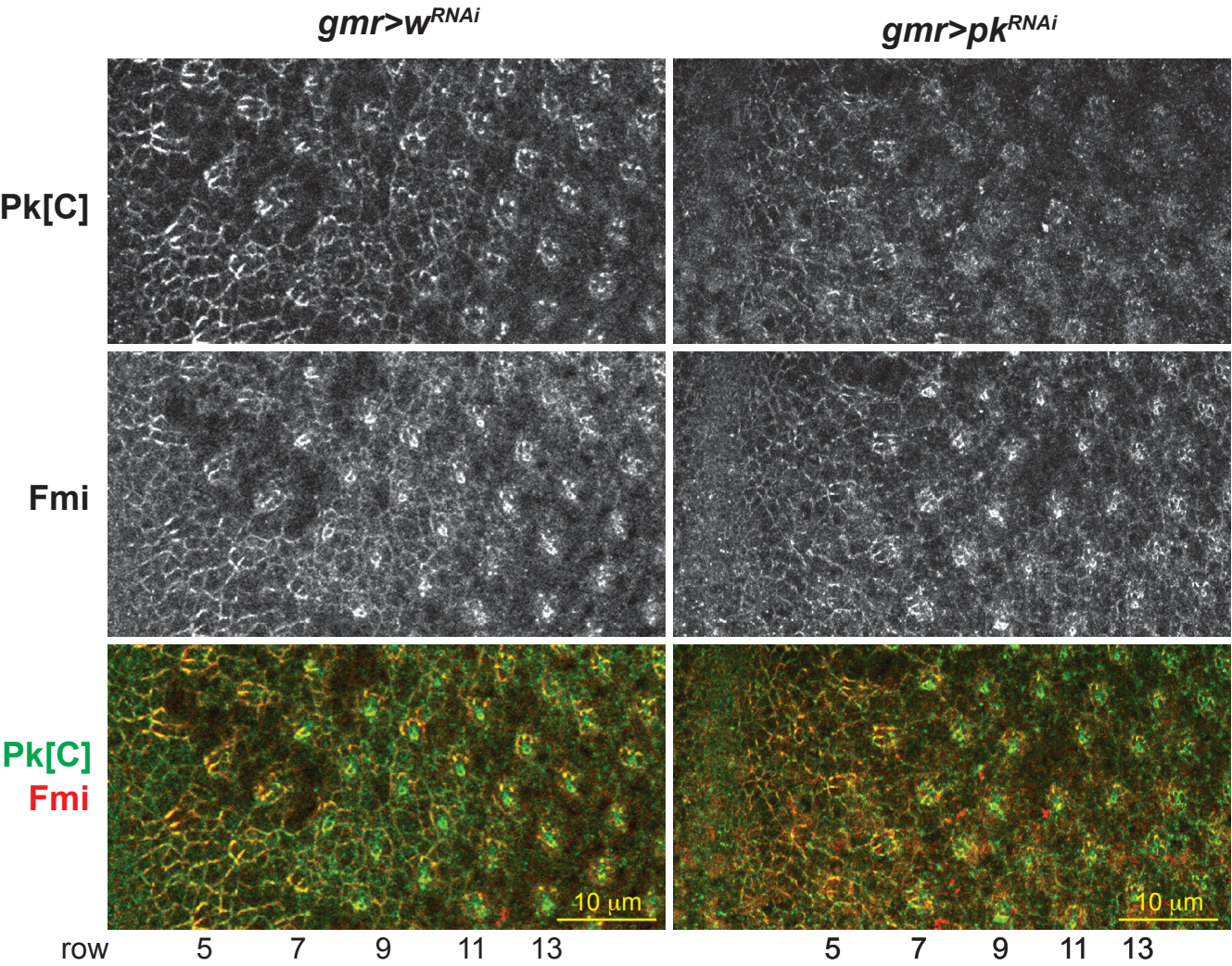

Supplement: S6 Fig — Control (GMR>wRNAi) and pk knockdown (GMR>pkRNAi) eyes labeled with anti-Pk[C] (green in merge) and anti-Fmi (red in merge) antibodies show knockdown of pk in older ommatidia in the GMR>pkRNAi eye. (PDF) [file pone.0262328.s006.pdf]

**A**

**Pk[C]**

**B**

**Fmi**

**C**

**Pk[C] Fmi**

**10  $\mu$ m**

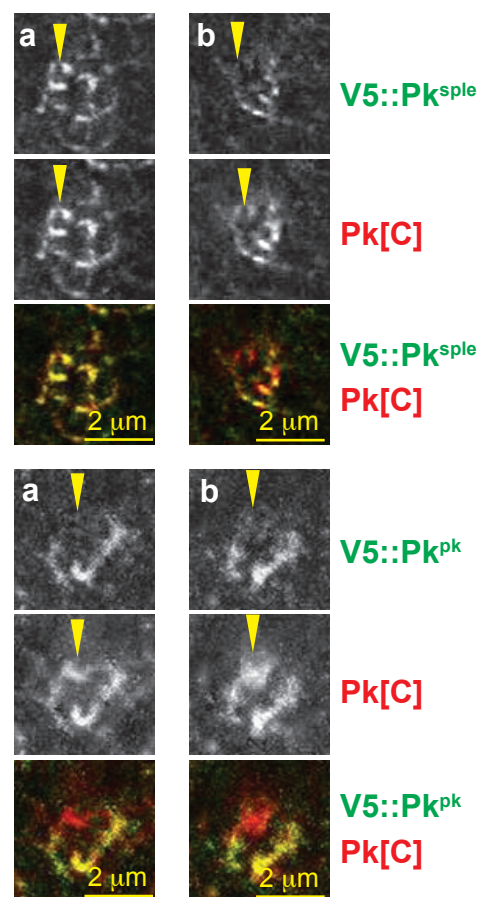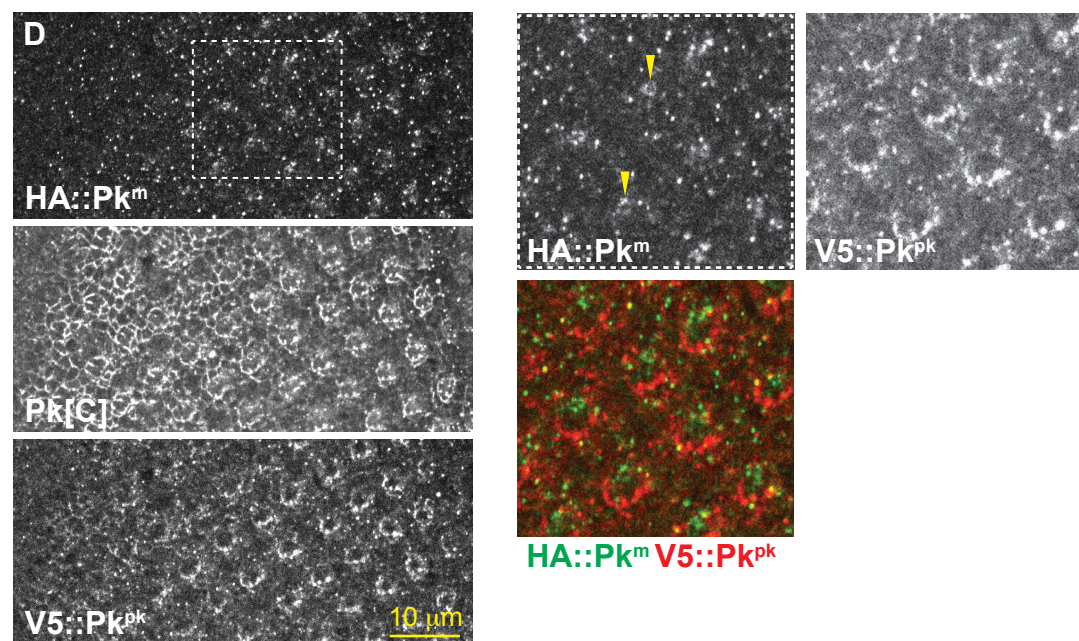

Supplement: S7 Fig — A. The Pk common antibody (Pk[C]) detects signal in R4 of older ommatidia, recognizable by their intense Fmi staining. B-C. V5::pksple and V5::pkpk eyes labeled for V5 and Pk[C] show absence of V5::Pksple and V5::Pkpk singal in R4’s of older ommatidia. V5::Pksple is still weakly detected in R4 at row 9 (Ba) but is absent from R4 by row 11 (Bb). V5::Pkpk is not detected in row 11 (Ca) or row 13 (Cb) ommatidia. D. A HA::pkm, V5::pkpk eye stained for HA, Pk[C] and V5. HA::Pkm is seen in older R4 cells and V5::Pkpk is detected in other cells but not in R4. The dotted region is enlarged on the right. (PDF) [file pone.0262328.s007.pdf]

Supplemental Figure 6-2

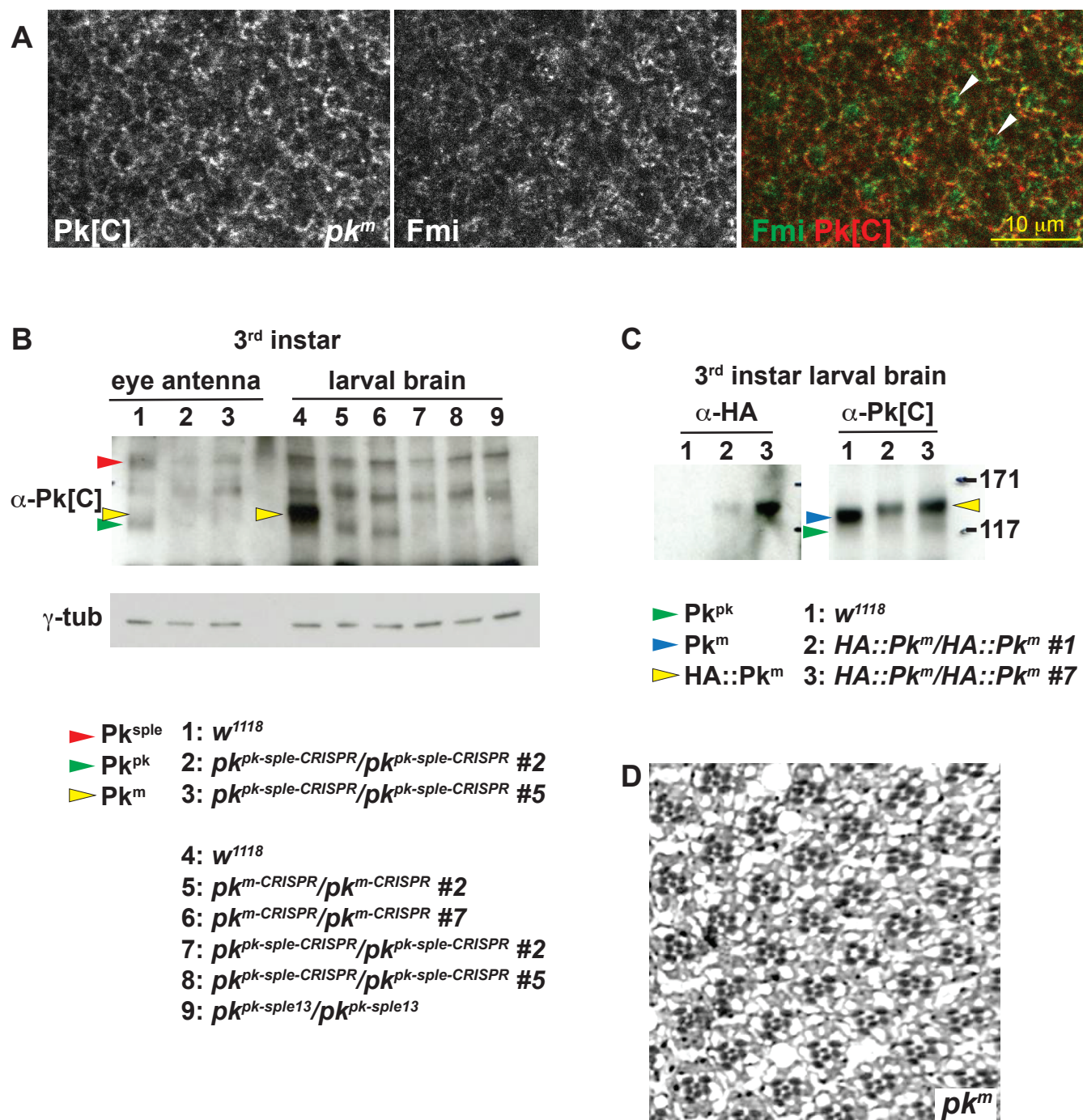

Supplement: S8 Fig — A. A pkm mutant eye stained with the Pk[C] common antibody and anti-Fmi shows that no Pk signal is detected in the R4 cells of late ommatidia (arrowheads). B. A Western blot from control eye and larval brain probed with the Pk[C] antibody detects little if any Pkm in eyes, but Pkm is the predominant isoform in brain. C. A Western blot from control and HA::Pkm larval brain probed sequentially with anti-HA and the Pk[C] common antibody shows detection of endogenous Pkm in control and HA-tagged Pkm in the tagged strains. The HA::Pkm #1 lane is slightly under-loaded relative to the other lanes, and uneven developing occurred in the anti-HA blot. Both HA::Pkm lines therefore express at similar levels to endogenous Pkm. D. A section of a pkm mutant eye shows normal polarity. (PDF) [file pone.0262328.s008.pdf]

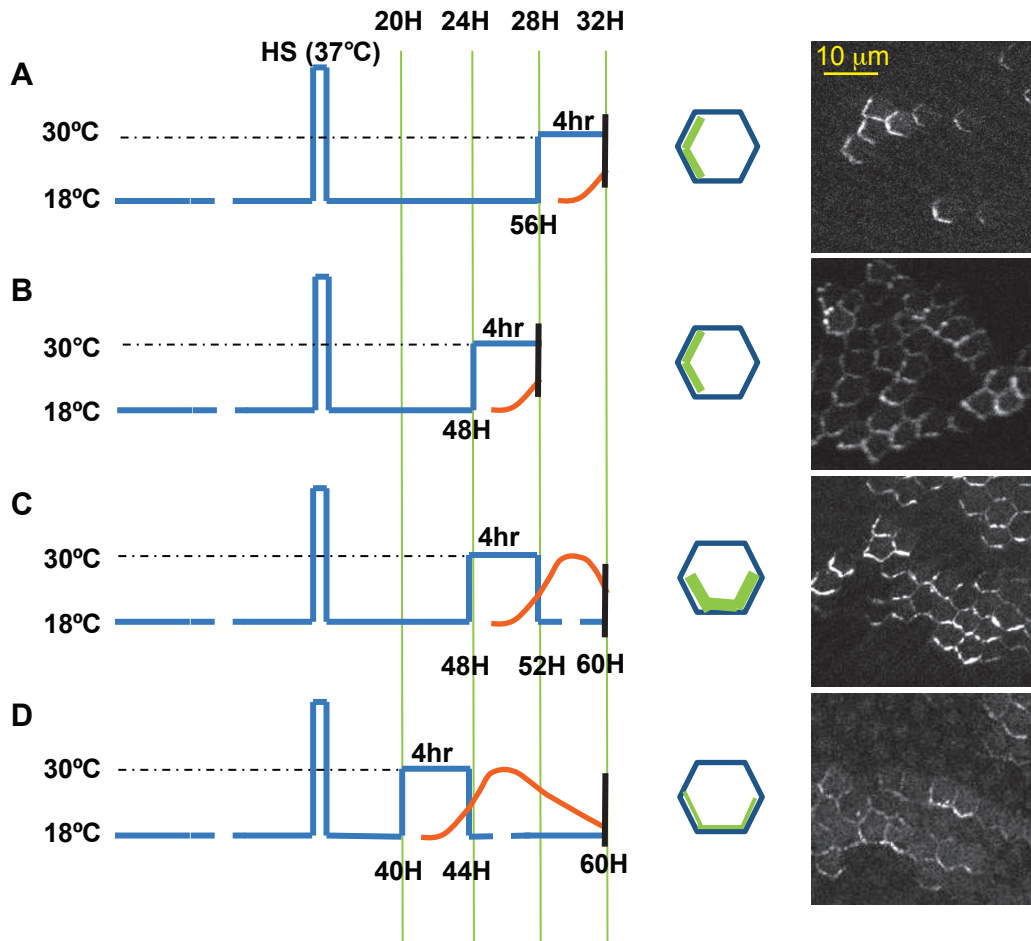

**E 28H wing**

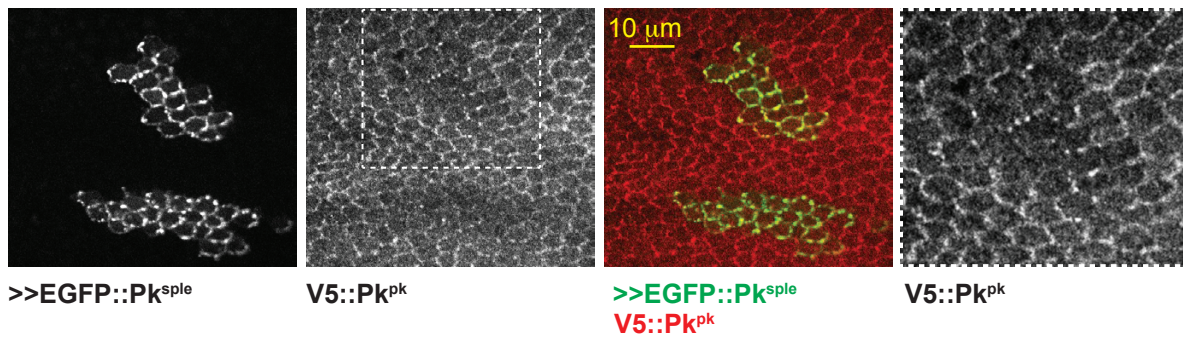

**F 40H wing**

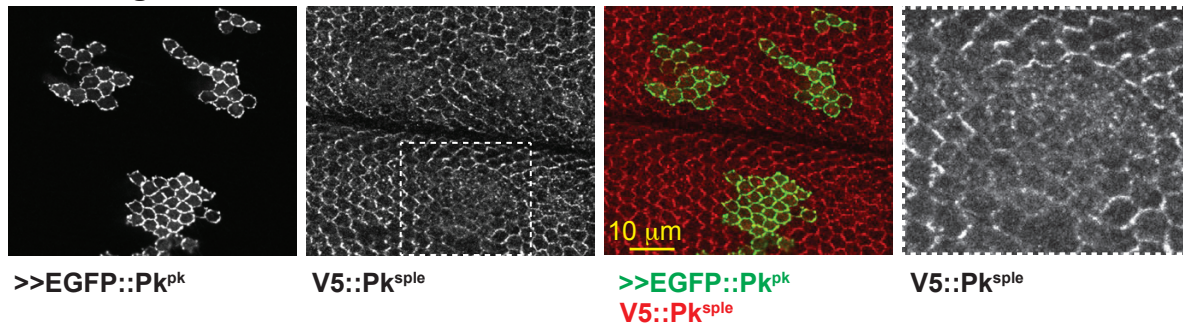

Supplement: S9 Fig — A-D. Pulsed expression of Pksple in clones in the wing, allowed to accumulate at the permissive temperature (30°) starting at the indicated times and allowed to accumulate or decay for varying times. Heat shocks at 37° induced clones. Flies were maintained at the restrictive temperature (18°) until expression was induced at 30° (blue lines). The expected protein accumulation is shown in red, and equivalent developmental time is shown at the top. Modest expression first shows that Pksple accumulates on the proximal sides of cells where Pkpk is already present (A-B). After accumulating to a higher level, Pksple takes over and polarity is reoriented with Pksple localizing to the posterior as expected from the Ds/Fj gradients (C-D; clones in the posterior wing). E. High level tagged Pksple clonal expression beginning in third instar in V5::Pkpk wings shows that high levels of Pksple out-compete and displace Pkpk from apico-lateral junctions at 28H after puparium formation. F. High level tagged Pkpk clonal expression beginning in third instar in V5::Pksple wings shows that high levels of Pkpk out-compete and displace Pksple from apico-lateral junctions at 40H after puparium formation, when Pksple is the predominant isoform [36]. 8 to 10 clones were analyzed for each condition and representative clones are shown. (PDF) [file pone.0262328.s009.pdf]
